# When Giants Turn Up: Sighting Trends, Environmental Influences and Habitat Use of the Manta Ray *Manta alfredi* at a Coral Reef

**DOI:** 10.1371/journal.pone.0046170

**Published:** 2012-10-03

**Authors:** Fabrice R. A. Jaine, Lydie I. E. Couturier, Scarla J. Weeks, Kathy A. Townsend, Michael B. Bennett, Kym Fiora, Anthony J. Richardson

**Affiliations:** 1 Biophysical Oceanography Group, School of Geography, Planning and Environmental Management, The University of Queensland, St Lucia, Australia; 2 Climate Adaptation Flagship, CSIRO Marine and Atmospheric Research, Ecosciences Precinct, Dutton Park, Australia; 3 School of Biomedical Sciences, The University of Queensland, St Lucia, Australia; 4 School of Biological Sciences, The University of Queensland, St Lucia, Australia; 5 Moreton Bay Research Station, The University of Queensland, Dunwich, Australia; 6 Watersports, Lady Elliot Island Eco Resort, PO Box 5026, Torquay, Australia; 7 Centre for Applications in Natural Resource Mathematics, School of Mathematics and Physics, The University of Queensland, St Lucia, Australia; Hokkaido University, Japan

## Abstract

Manta rays *Manta alfredi* are present all year round at Lady Elliot Island (LEI) in the southern Great Barrier Reef, Australia, with peaks in abundance during autumn and winter. Drivers influencing these fluctuations in abundance of *M. alfredi* at the site remain uncertain. Based on daily count, behavioural, weather and oceanographic data collected over a three-year period, this study examined the link between the relative number of sightings of manta rays at LEI, the biophysical environment, and the habitat use of individuals around the LEI reef using generalised additive models. The response variable in each of the three generalised additive models was number of sightings (per trip at sea) of cruising, cleaning or foraging *M. alfredi*. We used a set of eleven temporal, meteorological, biological, oceanographic and lunar predictor variables. Results for cruising, cleaning and foraging *M. alfredi* explained 27.5%, 32.8% and 36.3% of the deviance observed in the respective models and highlighted five predictors (*year*, *day of year*, *wind speed*, *chlorophyll-a concentration* and *fraction of moon illuminated*) as common influences to the three models. There were more manta rays at LEI in autumn and winter, slower wind speeds, higher productivity, and around the new and full moon. The winter peak in sightings of foraging *M. alfredi* was found to precede peaks in cleaning and cruising activity around the LEI reef, which suggests that enhanced food availability may be a principal driver for this seasonal aggregation. A spatial analysis of behavioural observations highlighted several sites around the LEI reef as ‘multi-purpose’ areas where cleaning and foraging activities commonly occur, while the southern end of the reef is primarily a foraging area. The use of extensive citizen science datasets, such as those collected by dive operators in this study, is encouraged as they can provide valuable insights into a species' ecology.

## Introduction

Understanding drivers of spatial distribution and habitat selection in large and highly mobile marine species is crucial for implementing effective management strategies, especially for those species with small population sizes and that are significantly impacted by fisheries. For free-ranging marine animals, drivers of movements are often difficult to elucidate and can be as diverse as the need to reproduce and maintain genetic diversity [1], [2], their respective physiologies [3]–[5] or the distribution and availability of a preferred food resource [6]–[8]. Obtaining daily data on the distribution and behaviour of many wide-ranging pelagic species is equally challenging. Recent advances in technology have provided an array of methodologies ranging from animal-attached sensors for collecting data on an animal's movements, behaviour, physiology and/or environment [9]–[13], through to more conventional and cost-effective approaches such as photographic-identification [14], [15] or multi-year observational records [16], [17]. While the former methodologies can prove costly and present several technical and logistical challenges [18]–[20], multi-year observational records of species are relatively easy to collect and can provide valuable insights into patterns of occurrence and behaviour of a target species at a specific site. Coupled with *in situ* environmental observations and appropriate statistical models, such datasets offer a great opportunity to explore and identify key drivers for the presence of a particular species at a specific site [e.g. 16], [17].

Manta rays are the largest batoid fishes in the world and rank amongst the largest plankton-feeding elasmobranchs, with a worldwide distribution in tropical and subtropical regions [20]–[22]. Manta rays are increasingly targeted by fisheries in several parts of the world, particularly due to the high value of their gill rakers that are used in ‘traditional’ Chinese medicine [22]. However, current understanding of the basic ecology of manta rays is relatively limited, particularly in terms of movements, habitat selection processes and drivers of their distribution [14], [20], [22]–[26]. With increasing fishing pressure and with global warming predicted to significantly impact reef systems, ocean dynamics and productivity in coming years [27]–[31], enhanced knowledge of manta ray critical habitats and migratory ecology is needed to ensure current management strategies are adequate and to help adapt these in the future.

**Figure 1:**
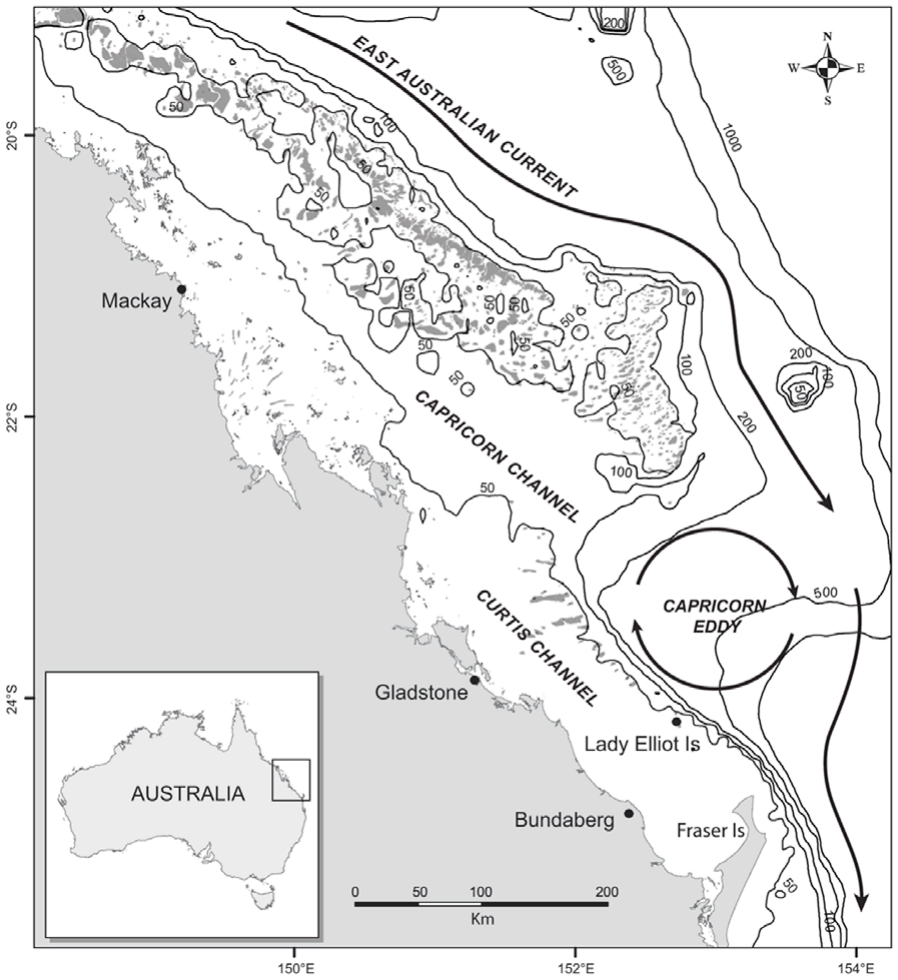
Bathymetric and oceanographic setting for Lady Elliot Island, southern Great Barrier Reef. Schematic map of the southern GBR showing location of LEI, bathymetry (full lines) and typical flow of the East Australian Current (bold arrows). Modified from [68].

In eastern Australia, the latitudinal range of the reef manta ray *Manta alfredi* is over 3,000 km and the species is commonly observed at various localities along the coastlines of northern New South Wales and Queensland, including waters of the Great Barrier Reef (GBR) [14], [32]. *Manta alfredi* is a common visitor to waters adjacent to Lady Elliot Island (LEI, 23°07′S, 152°43′E), a small coral cay located at the southernmost limit of the GBR Marine Park (Fig. 1), where it is found all year round, with peaks in abundance in autumn and winter [14]. However, reasons for the observed distributions of *M. alfredi* and fluctuations in numbers around LEI remain uncertain. Here we use a three-year observational dataset of daily *M. alfredi* numbers, behaviour and potential environmental drivers to identify key influences on the distribution, habitat use and abundance of the species at LEI. We use generalised additive models (GAMs) to examine the relationship between the abundance of cruising, cleaning and foraging *M. alfredi* at LEI from 2008–2011 and a set of temporal and environmental variables. We further examine habitat use around the LEI reef, compare results to current knowledge of *M. alfredi* and address further research directions that will improve current understanding of the behavioural ecology of this large planktivore.

## Methods

### Ethics

All necessary permits were obtained for this study. Work was conducted under permit from the GBR Marine Park Authority (G09/29853.1) and approval from the University of Queensland Animal Ethics Committee (SBMS/206/11/ARC). No animal was caught, handled or removed from its natural habitat for the purpose of this study.

### Manta ray sighting data

**Figure 2:**
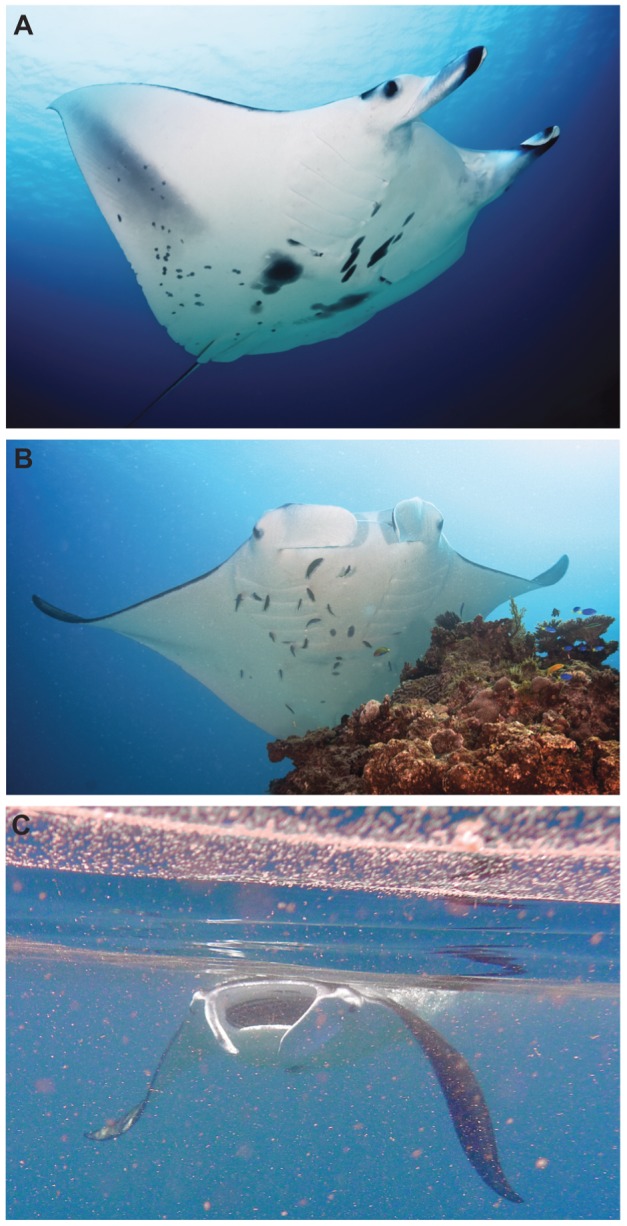
Manta ray behaviours. Photographs presenting the three main behaviours for *M. alfredi* observed around the LEI reef: (A) cruising (manta ray swimming with cephalic lobes rolled and mouth closed), (B) cleaning (manta ray at a ‘cleaning station’, maintaining a near stationary position atop a coral patch for several minutes while being cleaned by cleaner fishes), and (C) foraging (manta ray ram feeding - swimming against the tidal current with its mouth open and sieving zooplankton from the water).

Citizen science, whereby members of the public engage in collecting scientific data about species distribution or occurrence over long time spans, has been remarkably successful in advancing scientific knowledge over the years [33]–[36]. The manta ray sighting data analysed in this study were collected by KF and a team of volunteer SCUBA diving instructors and boat skippers from the local tourism operator on LEI. The number of manta rays sighted and the behaviour they displayed were recorded for each trip at sea made between May 2008 and May 2011, both at the surface via boat-based observations and underwater while on SCUBA. Manta ray sightings per trip observations are used here as a measure of the relative abundance of manta rays at the study site. Most trips lasted 120–150 minutes and the monitoring effort was fairly consistent throughout the study period, with 1,264, 1,387 and 1,226 boat hours logged for each year, respectively. For each of the 1,605 records analysed in this study, date, time, site, number of individuals sighted and the behaviour displayed were recorded on a standardised log sheet. The dataset included 443 records for the first year (27.6% of total), 554 (34.5%) for the second year and 608 (38.8%) for the third year, totalling 9,769 manta ray sightings around the LEI reef. Trip destinations and dive sites (with an average depth of ∼15 m) were primarily determined in relation to wind direction and speed, sea state and activities to be undertaken. Trips occurred in a variety of conditions, including large swells and with wind speeds of up to 50 km.h^−1^, and were only cancelled on a few occasions during exceptionally inclement weather, such as when Tropical Cyclone Ului hit the GBR in early March 2010. *Manta alfredi* sighted around the LEI reef were categorised into three main behaviours, depending on whether they were simply cruising (manta ray swimming with cephalic lobes rolled and mouth closed), cleaning (manta ray at a ‘cleaning station’, maintaining a near stationary position atop a coral patch for several minutes while being cleaned by cleaner fishes) or foraging (manta ray ram feeding – swimming against the tidal current with its mouth open and sieving zooplankton from the water) (Fig. 2). Each individual was only recorded under one behavioural category at a time; for example, if a manta ray was observed cruising and stopping at a cleaning station for several minutes before leaving the site, it was recorded as ‘cleaning’. Manta rays were recorded as ‘cruising’ when they did not display either cleaning or foraging behaviour during the time they were observed at the site.

### Environmental predictors

For each trip around the LEI reef, a suite of environmental variables was recorded (Table 1). Since sea temperature could influence the physiology and long-term behavioural strategies of many species, especially large ectothermic fishes [3], [37], [38], we measured sea temperature in the upper 15 m of the water column using a dive computer. Wind has been shown to affect the abundance of whale sharks *Rhincodon typus*, another large elasmobranch planktivore, in Western Australian waters [17]. Hence, *in situ* wind speed and direction data were obtained from an automated weather station on LEI (Australian Bureau of Meteorology station 039059). Wind data were recorded twice a day, at 09:00 and 15:00 and the closest time to each manta ray observation was used. Prevailing current direction was recorded since regional and local currents have previously been suggested to influence the occurrence, abundance and behaviour of other large planktivores at other localities [39], [40]. Current direction was categorized according to the two main current scenarios around the LEI reef: typically flowing in a southward direction during flood tides, and northward during ebb tides (KF *pers. obs.*). Tides have been shown to influence the behaviour of many reef species [41], [42], including manta rays in the northern GBR [32]. Therefore, we included two tidal predictors in the model: *time relative to high tide* and *tidal range*. Tidal data for LEI were obtained through the XTide Tide Prediction Server (http://www.mobilegeographics.com), which corrected NOAA's National Ocean Service tide data from the nearest port (Bundaberg) to LEI. Moon phase has been previously suggested to influence the behaviour and visitation patterns of some species to particular reefs [41], [43], [44], including manta rays [23]. Here we used the fraction of moon illuminated as a proxy for moon phase, since it allowed the examination of this predictor as a continuous variable. Data on the fraction of moon illuminated was sourced from the United States Naval Observatory (http://www.usno.navy.mil/USNO).

**Table 1:** List of explanatory variables used in this study for the period May 2008 to May 2011. Details include source and unit of measure for each continuous variable or category levels for categorical predictors (marked ^*^).

Explanatory variable	Units/Levels	Resolution	Source
Year^*^	year	N/A	Calendar
Day of year	day	1	Julian day calendar
Time of day	h	0.01	Eastern Standard Time
Sea temperature	°C	1	Dive computer
Wind speed	km.h^−1^	0.01	Weather station #039059 - Australian Bureau of Meteorology
Wind direction	°	0.5	Weather station #039059 - Australian Bureau of Meteorology
Current direction^*^	North, South	N/A	Observer
Chlorophyll-*a* concentration	mg.m^−3^	0.001	7-day means, MODIS data, 1 km resolution
Time relative to high tide	h	0.1	XTide Prediction Server
Tidal range	m	0.1	XTide Prediction Server
Fraction of moon illuminated	N/A	0.01	United States Naval Observatory

Zooplankton, the principal known food resource for manta rays, could not be readily collected for each observation throughout the three-year study period. However, since satellite-derived chlorophyll-*a* concentrations [chl-*a*] reflect the biomass of phytoplankton present in the upper photic layers of the water column [45], and since there is often high zooplankton biomass in areas of high phytoplankton biomass [46]–[50], [chl-*a*] was used as an indirect proxy for local productivity despite the recognised temporal lag between phytoplankton and zooplankton blooms [48], [51]. We used ocean colour data derived from the Moderate Resolution Imaging Spectroradiometer (MODIS; modis.gsfc.nasa.gov) to derive [chl-*a*] in the waters adjacent to LEI. Weekly mean [chl-*a*] images were generated at 1 km^2^ spatial resolution for the period 2008–2011, using the standard [chl-*a*] algorithm [45]. Data were then extracted from a 3×3 pixel area in sufficiently deep waters (i.e. 32 m depth) directly adjacent to the LEI reef so as to avoid bottom contamination of the satellite signal. Each sighting record was then matched to the corresponding weekly [chl-*a*] value.

### Modelling approach

We used generalised additive models (GAMs) [52], [53] as an exploratory data analysis tool for elucidating functional forms of relationships between sightings of cruising, cleaning and foraging manta rays per trip and the set of selected predictors. GAMs can be useful for interpreting ecological relationships, as they are able to fit non-parametric functions to estimate the form of the relationship between response and predictors without imposing *a priori* limitations on its form [54], [55]. GAMs have been increasingly used to explore relationships between the abundance and distributions of marine species and the surrounding environment [56]–[60]. GAMs are known to perform well with presence-only datasets when species absence data cannot be collected from a systematic stratified survey [61], as in the present study. The GAM used in this study followed the form:
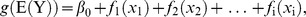
where *g* is a link function, *β* is a constant and *f*_i_(*x*_i_) is a smoothing function applied to each continuous variable.

We implemented one GAM for each manta ray behaviour, namely cruising, cleaning and foraging, observed around the LEI reef. For each model, our response variable was a count variable of sightings per trip. We used 11 predictors, some of which were continuous and some categorical (Table 1), with no strong cross-correlations between predictors (Table S1). Each GAM was implemented using the gam package in R [62] and used a Poisson error structure and log link function to fit the response. Predictor variables listed in Table 1 were fitted based on the Likelihood-ratio test of comparing the full model and the model omitting the respective predictor [63], [64]. As such, only variables found to be significant (p<0.05) were included in each model. One of the predictors, *day of year*, was fitted with a harmonic polynomial to ensure continuity between beginning and end of the year signals. Graphical output of each GAM provides a visual representation of relationships between the response and predictors. The *y*-axis is a relative scale, so that the range of the values displayed represents the importance of each variable. The shape and significance of the relationship of each variable allowed us to describe how each predictor influenced the relative abundance of cruising, cleaning and foraging manta rays at LEI.

## Results

### Cruising manta rays

**Figure 3:**
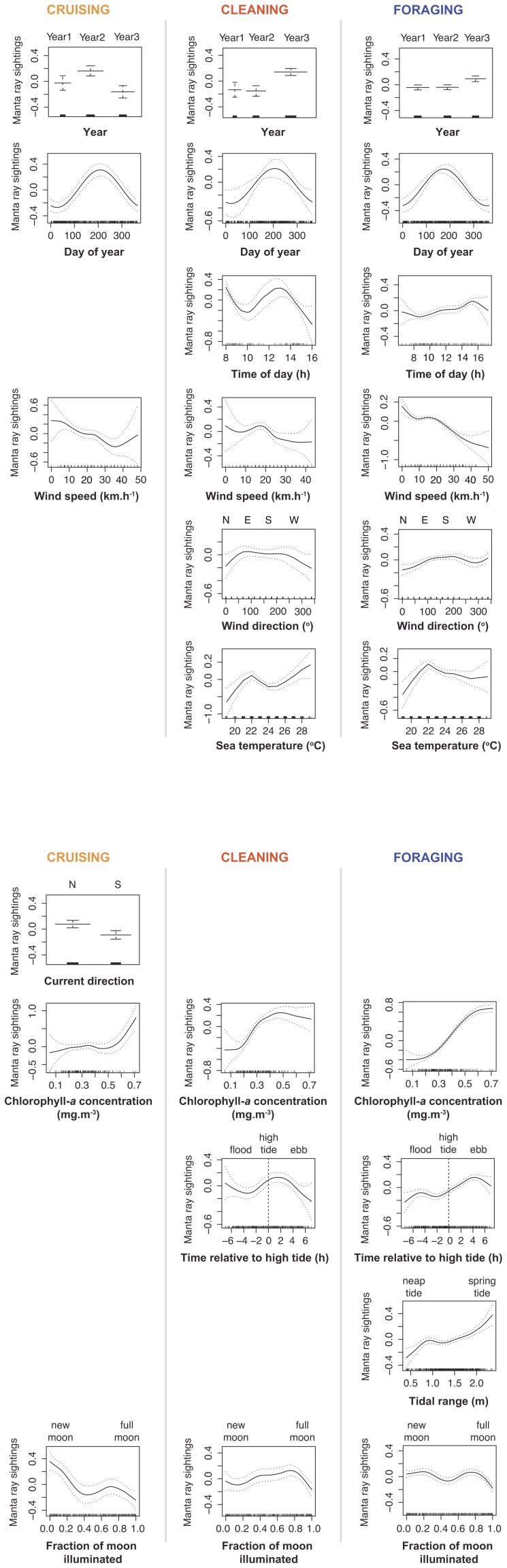
Generalised additive model output for each manta ray behaviour observed at LEI. Results for the functional relationships between sightings of cruising, cleaning and foraging *M. alfredi* per trip around the Lady Elliot Island reef and the set of temporal and environmental predictors. Each column presents a different behaviour and each row presents results for the influence of a predictor to each behavioural activity, where significant. For each plot, the *y*-axis is a relative scale, and its magnitude reflects the importance of each variable. Dashed lines and error bars represent 95% confidence intervals.

The GAM for cruising manta rays was the weakest of the three models, comprising six predictors that explained 27.5% of the deviance observed. *Fraction of moon illuminated*, *day of year*, *chlorophyll-a concentration*, and *wind speed* showed the strongest effects on the relative abundance of cruising manta rays at LEI (Table 2). Graphical output revealed that numbers of cruising manta rays at the study site were highest during Year 2 and lowest during Year 3, and typically peaking between days 170–250 (mid-June to early-September) (Fig. 3). There were more sightings of cruising manta rays with slower wind speeds, during northward currents, with high [chl-*a*], and around the new moon.

**Table 2:** Summary of the generalised additive model for assessing the influence of each predictor to the relative abundance manta rays (*M. alfredi*) exhibiting cruising, cleaning and foraging behaviour at LEI.

	CRUISING				CLEANING				FORAGING			
Term added to model	Deviance	d.f.	p (χ^2^)	% Dev. Exp.	Deviance	d.f.	p (χ^2^)	% Dev. Exp.	Deviance	d.f.	p (χ^2^)	% Dev. Exp.
Year	16.06	2	<0.05	**2.4**	19.52	2	<0.05	**1.6**	15.61	2	<0.05	**0.3**
Day of Year	42.47	2	<0.05	**6.4**	7.22	2	<0.05	**0.6**	84.60	2	<0.05	**1.5**
Time of day	–	–	–	–	40.56	1	<0.05	**3.2**	54.52	1	<0.05	**0.9**
Sea temperature	–	–	–	–	68.31	1	<0.05	**5.4**	93.67	1	<0.05	**1.6**
Wind speed	23.85	1	<0.05	**3.6**	16.32	1	<0.05	**1.3**	152.15	1	<0.05	**2.6**
Wind direction	–	–	–	–	10.20	1	<0.05	**0.8**	33.90	1	<0.05	**0.6**
Current direction	7.07	1	<0.05	**1.1**	–	–	–	–	–	–	–	–
Chlorophyll-*a* concentration	25.73	1	<0.05	**3.9**	56.63	1	<0.05	**4.5**	525.45	1	<0.05	**9.0**
Time relative to high tide	–	–	–	–	19.54	1	<0.05	**1.6**	96.90	1	<0.05	**1.7**
Tide Range	–	–	–	–	–	–	–	–	46.06	1	<0.05	**0.8**
Fraction of moon illuminated	44.19	1	<0.05	**6.7**	27.72	1	<0.05	**1.7**	54.90	1	<0.05	**0.9**
**Full model**	660.13	483	–	**27.5**	1255.09	436	–	**32.8**	5806.49	683	–	**36.3**

The significance (p-value) of each term was based on the Likelihood-ratio test of comparing the full model and the model omitting the respective predictor, and only significant predictors were included in each model. The percentage of deviance explained (% Dev. Exp.) represents the importance of each predictor.

### Cleaning manta rays

The GAM for cleaning manta rays explained 32.8% of the deviance observed in sightings of manta rays cleaning around the LEI reef. The model comprised nine predictors, of which *sea temperature*, *chlorophyll-a concentration*, *time of day*, *fraction of moon illuminated*, *time relative to high tide* and *year* were strongest (Table 2). Graphical output revealed numbers of manta rays cleaning at LEI were higher during Year 3, typically peaking between days 170–250 (mid-June to early-September), and lower between 09:00–11:00 in the morning and after 14:00 in the afternoon (Fig. 3). Sightings of cleaning manta rays showed a slightly decreasing trend as wind speeds increased and there were fewer sightings during north and north-west winds. Sightings of manta rays cleaning increased with warmer sea temperatures and higher [chl-*a*]. *Time relative to high tide* highlighted highest cleaning activity at the site around high tide and decreasing 3–4 h after high tide, and the *fraction of moon illuminated* showed lower sightings of cleaning individuals around new and full moons.

### Foraging manta rays

The GAM for foraging manta rays was the strongest of the three models, explaining up to 36.3% of the deviance observed in sightings of foraging manta rays. The model included 10 predictors, with *chlorophyll-a concentration*, *wind speed*, *time relative to high tide*, *sea temperature* and *day of year* showing the strongest effects (Table 2). Graphical output showed more sightings of foraging manta rays during Year 3, typically peaking between days 130–210 (mid-May to late-July) and increasing throughout the day to peak in the late afternoon (Fig. 3). Sightings of foraging individuals strongly decreased as wind speed increased, and with lower numbers during northerly winds. *Sea temperature* highlighted a strong peak in sightings of foraging manta rays for temperatures between 21–23°C. There was a strong positive linear relationship between the number of sightings of foraging individuals and [chl-*a*]. Sightings also typically increased with tidal range and throughout the tidal cycle, peaking around 4 h after high tide. *Fraction of moon illuminated* highlighted higher sightings of foraging manta rays around the new moon and preceding the full moon.

### Spatial occurrence and habitat use

**Figure 4:**
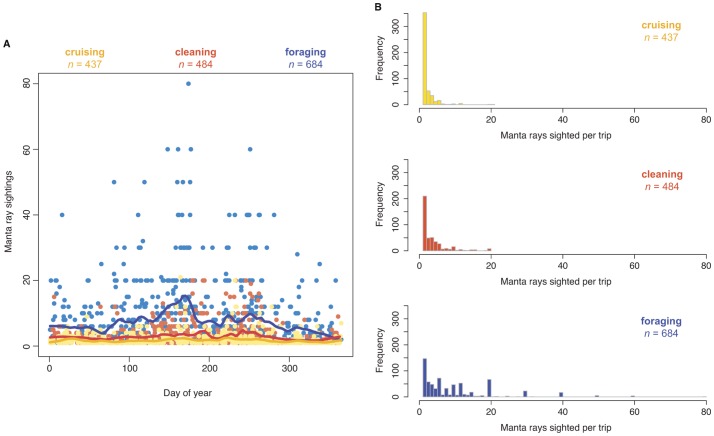
Sighting trends for cruising, cleaning and foraging manta rays at Lady Elliot Island. (A) Manta ray sighting records throughout the year for each behaviour; dots indicate data records and full lines show overall temporal trend via locally weighted scatterplot (loess) smoothing. (B) Frequency distributions of numbers of cruising, cleaning and foraging manta rays sighted per trip.

Further analysis revealed that foraging activity showed the strongest variability in numbers of manta rays sighted, with large aggregations of foraging *M. alfredi* more common between days 90 to 250 (April to September) (Fig. 4a). Frequency distributions of manta ray numbers per observation revealed that while most observations of cruising and cleaning *M. alfredi* consisted of relatively few animals, foraging *M. alfredi* were commonly observed in larger groups (Fig. 4b), with up to 80 individuals counted on one occasion.

**Figure 5:**
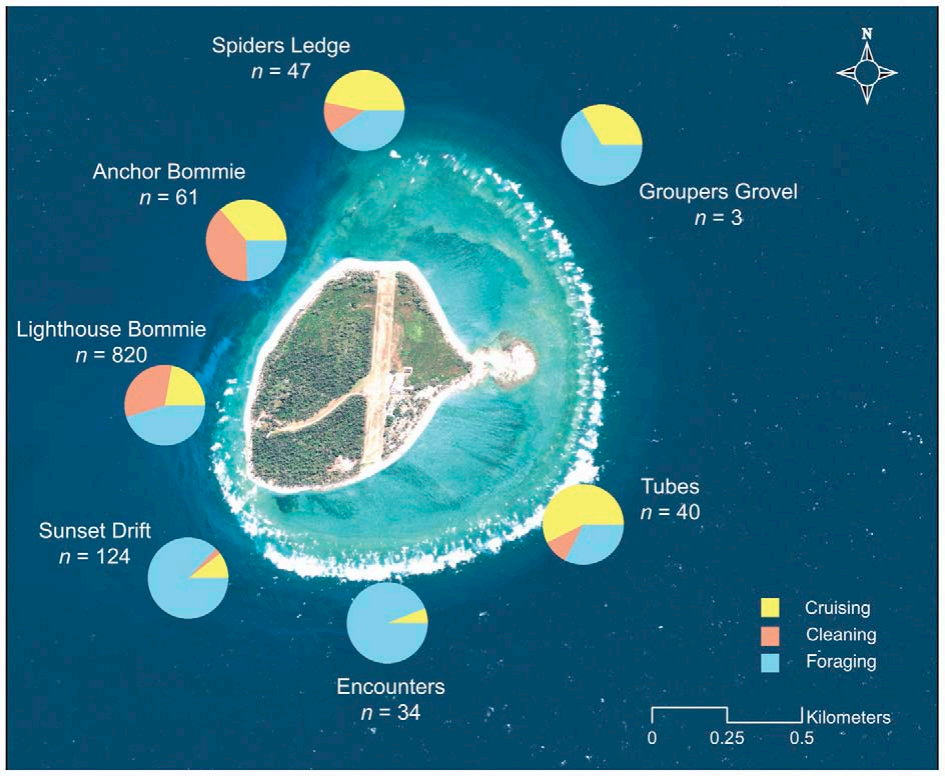
Habitat use for manta rays at Lady Elliot Island. Map of Lady Elliot Island showing total observations of *M. alfredi* at seven sites around the reef, between May 2008 and May 2011. Pie charts indicate percent activity at each site for cruising, cleaning and foraging manta rays. High-resolution image of LEI obtained from the Quickbird orbiting satellite (Geoimage Pty Ltd., www.geoimage.com.au) and data overlaid using ArcGIS 10 (ESRI, www.esri.com/software/arcgis).

A spatial examination of behavioural data, using observations made at seven sites located around the margins of the LEI reef, provided further insights into the habitat use of *M. alfredi* at LEI (Fig. 5). The majority of observations (51% of total records) occurred at one site, ‘Lighthouse Bommie’, a popular ‘cleaning station’ for *M. alfredi*. While cleaning manta rays accounted for 32.5% of observations at this site, foraging animals were also extremely common (45.4%). Further sites on the western side of the reef displayed similar relationships, where both important cleaning and foraging activity were recorded. By contrast, other sites were characterised by a single dominant activity. For example, sites such as ‘Sunset Drift’ and ‘Encounters’, both located around the southern end of the LEI reef, showed high foraging activity (respectively 87% and 94% of observations at these sites) and very few manta rays observed cleaning or cruising.

## Discussion

The GAM modelling approach, which analysed the number of sightings for each behaviour, highlighted five predictors (i.e. *year*, *day of year*, *wind speed*, *chlorophyll-a concentration* and *fraction of moon illuminated*) as common influences on the relative abundance of cruising, cleaning and foraging manta rays at LEI. While no conclusions can be drawn with regards to the observed inter-annual variations in manta ray sightings at LEI due to the limited study period, the four remaining predictors common to the three models are major influences on the overall relative abundance of *M. alfredi* at the site, with numbers of manta rays typically peaking in autumn and winter, decreasing with increased wind speeds, increasing with increased productivity and higher around the new and full moon.

### Seasonality

The *day of year* predictor highlighted a strong seasonal peak in the relative abundance of *M. alfredi* at LEI, with more manta rays sighted between early-May to mid-August. This trend is consistent with results from Couturier *et al.* (2011), who used photographic-identification to determine the presence of individually recognisable *M. alfredi* at LEI and found that more manta rays were present during autumn and winter (i.e. peaking in June/July) [14]. Moreover, while *M. alfredi* exhibits some degree of site affinity and is present year round at several localities around the globe, seasonal peaks in animal numbers at these sites have also been observed [20], [22]–[24], [65]. Reasons for such seasonal aggregation patterns remain uncertain, although increased local productivity and subsequent increased food availability during manta ray peak aggregation periods have been noted [20], [22], [24], [66].

LEI is located seven kilometres away from the edge of the continental shelf on the southern extent of the Capricorn Wedge, a major inflection in the shelf (Fig. 1). The oceanography of the region is dominated by the southward-flowing East Australian Current that drives warm, nutrient-poor surface waters along the continental shelf [67]. An important oceanographic feature, and potentially important driver for the observed patterns in manta ray abundance at LEI, is the nearby-forming cyclonic Capricorn Eddy (Fig. 1) [68]. The Eddy is known to trigger upwelling of cool, nutrient-rich, sub-surface waters onto the shelf and around the Capricorn-Bunker reefs [68]–[71]. Although the periodicity of such intrusions of nutrient-rich waters onto the shelf has not yet been thoroughly documented for this region, early oceanographic observations identified frequent shelf-break upwelling and a mesoscale cyclonic eddy (the Capricorn Eddy) more apparent between June and December [69]–[71]. Further, the *chlorophyll-a concentration* predictor, used here as a proxy for local productivity, revealed that more manta rays were observed at the site in high [chl-*a*] scenarios. Nutrient enrichment via upwelling intrusions is well-known to initiate phytoplankton blooms and large increases in zooplankton [72], [73]. As such, the observed seasonal peak in abundance of *M. alfredi* at LEI may relate to the seasonality in regional oceanographic dynamics and subsequent enhanced supply of productive waters to the LEI reef.

### Diurnal visitations and habitat use

Motivations behind manta ray visitations to particular inshore localities can be diverse; while foraging manta rays are commonly observed at various sites around the globe [20], [22]–[26], cleaning is another important activity and some individuals have been observed to regularly visit inshore reefs and spend considerable amounts of time at dedicated ‘cleaning stations’ [23], [32], [74]–[76]. Moreover, the timing of visitations often relates to a particular activity [23], [32]. Our results revealed that while sightings of foraging manta rays typically increase throughout the day at LEI, visitations to cleaning stations were highest in the early morning and early afternoon, although the confidence limits are broad here because of relatively few data collected before 08:00 and after 16:00. Similar manta ray visitation patterns have been observed at other shallow coastal sites around the world such as the Komodo Marine Park, Indonesia, where manta rays show a clear diurnal activity pattern and are not present at night [23], [74], [77]. It is unknown where they go when leaving these sites, although it has been suggested that they may move offshore or to deeper waters at night [23], [74], [77].

The behaviour of manta rays was not uniform across all seven discrete sites around the LEI reef that were monitored. Along the western side of the reef several sites appear to be important for cleaning, but with cruising and foraging individuals also commonly observed. However, some other sites at the southern end of the LEI reef appear to be used primarily for foraging, as opposed to cleaning or cruising, with large aggregations of manta rays regularly observed feeding on dense zooplankton patches concentrated along tidal slicks.

Time- and site-specific increases in manta ray numbers, along with particular behaviours in several other marine species, are commonly attributed to tidal and lunar dynamics [23], [32], [43], [78]–[80]. Here, sightings of cleaning *M. alfredi* were highest around high tide and peaking within the first hours of the ebb tide, which is similar to observations at another cleaning station in the northern GBR [32]. By contrast, foraging activity increased throughout the tidal cycle to peak four to five hours on the ebb tide, which typically corresponds to strongest northerly currents at the site (KF *pers. obs*.). Tidal currents around the LEI reef typically shift 180 degrees with each tidal change and can be relatively strong (∼5 knots). In addition, there were highest abundances of foraging manta rays during spring tides, when tidal intensity and water exchange are the greatest [81], [82]. The *fraction of moon illuminated* predictor indicated higher sightings of cleaning manta rays during the first and third quarters of the moon, while cruising individuals were highest around the new moon and sightings of foraging *M. alfredi* greater around the new and preceding the full moon, precisely timed with spring tides. Similar patterns were observed in the Komodo Marine Park where manta ray visitations were highest when tidal intensity was greatest and precisely timed with new and full moons [23]. Local physical processes – especially tides, bathymetry and water currents – are well-known drivers of the concentration of zooplankton in specific areas of reefs [83]–[85]. It is thus likely that tidal dynamics around the LEI reef influence the spatial distribution of zooplankton by favouring the convergence and concentration of prey items around the southern end of the reef, and therefore attracting high numbers of *M. alfredi* to forage this area.

### Conclusions

This study examined trends of inter-annual, seasonal and diurnal variability in the relative abundance of cruising, cleaning and foraging *M. alfredi* at LEI and identified a set of key environmental drivers. Local productivity, winds, sea temperatures, and tidal processes all significantly influence the occurrence of manta rays at the site. Based on our observations, *M. alfredi* visited the LEI reef for both cleaning and foraging purposes, although courtship behaviour has also occasionally been observed at the site [14]. Manta ray foraging activity around the LEI reef fluctuated throughout the year, and peaked in winter. While *M. alfredi* occurs year-round at LEI, highest abundances are recorded in winter. We hypothesise that enhanced food availability during this time of the year is a principal driver of the observed seasonal aggregation, and that tides of greater intensity drive greater amounts of nutrient and plankton-enriched upwelled waters onto the shelf, ultimately influencing *M. alfredi* abundance at the site. A detailed investigation of local fine-scale dynamics relative to tidal processes, currents and zooplankton supply is currently underway, which may provide further insights into the ecology of these large planktivores and drivers for their presence at particular inshore localities. Such data are currently lacking for most manta ray aggregation sites around the globe. Although our results here indicate strong spatial distribution patterns of *M. alfredi* around the LEI reef, it is necessary to highlight the fact that our survey presents an inevitable bias associated with surveying only one side of the reef and one site at a time. Hence, further examination of occurrence patterns and residency times using a continuous sampling approach such as acoustic telemetry could aid in further understanding how manta rays use the LEI reef.

*Manta alfredi*, currently classified as Vulnerable on the IUCN Red List of Threatened Species [86], is the largest planktivorous fish that feeds regularly within GBR waters. Because large elasmobranch planktivores are ectotherms and feed primarily on zooplankton, which responds rapidly to changes in the surrounding environment [31], [50], *M. alfredi* is inevitably influenced by environmental variation. With increased fishing pressure in other parts of the world, and especially in waters neighbouring Australia [22], [87], and with climate change underway, identifying key environmental influences is necessary to improve management of the species and its habitat. Here we show that visitations and relative abundances of manta rays at LEI are related to a set of key behavioural, temporal and environmental factors. We believe that similar studies conducted at other manta ray aggregation sites around the globe will help refine current understanding of manta ray ecology.

## Supporting Information

Table S1 **Correlation matrix for continuous predictors included in the generalised additive models.** Each value is indicative of the degree of cross-correlation between predictors.
(PDF)
