# Supplementary material for: When Giants Turn Up: Sighting Trends, Environmental Influences and Habitat Use of the Manta Ray Manta alfredi at a Coral Reef
Source: PLoS One. 2012 Oct 3;7(10):e46170. doi: 10.1371/journal.pone.0046170 (PMC3463571; doi:10.1371/journal.pone.0046170)
Supplement: Table S1 — Correlation matrix for continuous predictors included in the generalised additive models. Each value is indicative of the degree of cross-correlation between predictors. (PDF) [file pone.0046170.s001.pdf]

	Day of year	Time of day	Wind speed	Wind direction	Sea temperature	Time to high tide	Tidal range	Moon illumination	Chlorophyll *a*
**Day of year**	1	-0.007	-0.035	0.023	-0.507	-0.021	0.051	0.083	0.015
**Time of day**	-0.007	1	-0.041	-0.06	0.107	-0.018	0.024	0.02	-0.045
**Wind speed**	-0.035	-0.041	1	0.01	0.088	0.011	0.038	0.06	-0.096
**Wind direction**	0.023	-0.06	0.01	1	-0.148	-0.067	-0.041	0.05	0.164
**Sea temperature**	-0.507	0.107	0.088	-0.148	1	0.021	0.001	-0.058	-0.312
**Time to high tide**	-0.021	-0.018	0.011	-0.067	0.021	1	-0.064	-0.012	0.033
**Tidal range**	0.051	0.024	0.038	-0.041	0.001	-0.064	1	-0.069	-0.094
**Moon illumination**	0.083	0.02	0.06	0.05	-0.058	-0.012	-0.069	1	-0.036
**Chlorophyll *a***	0.015	-0.045	-0.096	0.164	-0.312	0.033	-0.094	-0.036	1
